# Diagnostic Utility of Procalcitonin and Lactate Determination in Cerebrospinal Fluid for the Diagnosis of Neonatal Meningitis

**DOI:** 10.3390/jcm14020414

**Published:** 2025-01-10

**Authors:** Anna Borowiak, Krzysztof Safranow, Angela Sarna, Beata Łoniewska

**Affiliations:** 1 Department of Neonatal Diseases, University Clinical Hospital No. 2, Powstańców Wielkopolskich 72 Street, 70-111 Szczecin, Poland; beata.loniewska@pum.edu.pl; 2 Department of Biochemistry and Medical Chemistry, Pomeranian Medical University, Powstańców Wielkopolskich 72 Street, 70-111 Szczecin, Poland; krzysztof.safranow@pum.edu.pl; 3 Department of Laboratory Medicine, University Clinical Hospital No. 2, Powstańców Wielkopolskich 72 Street, 70-111 Szczecin, Poland; angi420@interia.pl; 4 Neonatology and Neonatal Intensive Care Clinic, Pomeranian Medical University, Siedlecka 2 Street, 72-010 Szczecin, Poland

**Keywords:** neonatal meningitis, cerebrospinal fluid, procalcitonin, lactate

## Abstract

**Objectives**: The diagnosis of meningitis is based on microbiological analysis of the cerebrospinal fluid, and the evaluation of cytosis and biochemical parameters such as protein and glucose levels. Sometimes when there is a traumatic lumber puncture, the cerebrospinal fluid is bloody, which makes it difficult to diagnose. The objective of the study was to examine the performance of cerebrospinal fluid (CSF) procalcitonin (PCT) and lactate as potential markers for the diagnosis of meningitis in neonates. **Methods**: 110 neonates who qualified for lumbar puncture were enrolled in the study. On the basis of CSF analysis, the neonates were classified into two groups: the meningitis group (*n* = 33) and the non-meningitis group (*n* = 77). PCT and lactate in CSF and established CSF parameters were recorded. **Results**: Median CSF PCT level was significantly higher in the meningitis group compared to non-meningitis: 0.93 (0.39–1.59) vs. 0.34 (0.195–0.74) ng/mL, *p* < 0.000001. Median CSF lactate level was significantly higher in the meningitis group compared to non-meningitis: 3.1 (2.27–3.96) vs. 1.78 (1.38–3.19) mmol/L, *p* < 0.001. At a cutoff of 0.35 ng/mL, CSF PCT had a sensitivity of 82% and specificity of 52% in the diagnosis of meningitis (AUC = 0.7). At a cutoff of 2.07 mmol/L, CSF lactate had a sensitivity of 84% and specificity of 60% in the diagnosis of meningitis (AUC = 0.701). **Conclusions**: Concentrations in CSF of PCT and lactate in neonates with meningitis are significantly higher than in the non-meningitis group. None of the biochemical indicators studied met the criteria for a marker for the diagnosis of meningitis as a single indicator.

## 1. Introduction

Meningitis is a life-threatening disease, defined as infection and inflammation of the meninges, subarachnoid space, and brain vasculature [1]. Meningitis in the neonatal group occurs much more frequently than in other age groups. The incidence of neonatal meningitis ranges from 0.21 per 1000 live births, with mortality ranging from 10 to 15% in developed countries, to 6.1 per 1000 live births, with a mortality of 40–58% in developing countries [2,3,4,5]. Neonatal meningitis should be diagnosed urgently and precisely and delaying its diagnosis and treatment can result in death or irreversible neurological consequences [1,2]. Positive cerebrospinal fluid (CSF) culture is the gold standard for the diagnosis of meningitis but has low sensitivity. Due to the lack of typical clinical symptoms of meningitis, CSF is usually collected for examination after the initiation of antibiotic therapy [3]. The preliminary result of this test is possible after 24–48 h. Therefore, in newborns demonstrating clinical symptoms, especially if they are accompanied by abnormalities in blood laboratory tests indicating the progression of infection, the diagnosis of meningitis is often made on the basis of the result of a general CSF examination, especially an evaluation of cytosis, protein, and glucose values [6,7,8,9,10]. Multiple studies have provided normative ranges for CSF parameters in term and preterm infants and described changes with advancing postnatal age, as well as in special circumstances, such as traumatic lumbar puncture and previous antibiotic administration [8,9,10]. Researchers are still looking for some biochemical indicators in CSF that will be helpful in diagnosing meningitis. Among them are lactate and PCT [11,12,13,14,15,16,17,18,19,20,21,22]. Studies that have attempted to determine lactate and PCT levels in the CSF mainly involve adults and children over 28 days of age and have shown to be a reliable marker in the diagnosis of meningitis and in differentiating between viral and bacterial etiologies of meningitis. However, despite years of work, an optimal “cutoff point” for lactate and PCT concentration in CSF has still not been established that can be considered binding for the diagnosis of meningitis in individuals beyond the neonatal period.

The significance of lactate and PCT determination in CSF in neonatal meningitis has not been widely studied to date and involves a small number of cases [23,24,25,26,27,28]. The purpose of this study is to analyze the diagnostic utility of selected biochemical parameters: PCT and lactate of cerebrospinal fluid in the diagnosis of meningitis in newborns, to define a cutoff for these markers in CSF that can be called significant in diagnosing meningitis.

## 2. Materials and Methods

This prospective, observational cohort study was conducted in the Department of Neonatal Diseases of the University Clinical Hospital No. 2 PUM in Szczecin in 2019–2021. The study included neonates who qualified for lumbar puncture. This included all neonates with early-onset sepsis and late-onset sepsis, with neurological manifestations and with a lack of improvement in general condition, despite treatment with antibiotics. Babies with severe congenital malformations and coagulopathy were excluded. Neonates were not excluded due to prior antibiotic use or traumatic lumbar punctures. The clinical signs that determined the decision to perform lumbar puncture, found in the study group of newborns, are summarized in Table 1. Investigations conducted on CSF were culture, pleocytosis, concentration of protein, sugar, PCT, and lactate (Table 2). After analyzing the result of the cerebrospinal fluid examination, meningitis was diagnosed or excluded. The following criteria for the diagnosis of meningitis based on the result of the CSF examination were adopted: a positive result from microbiological examination of CSF or at least two abnormal values in the total CSF examination:

- Cytosis more than 32/µL;
- Protein concentration of more than 120 mg/dL;
- Glucose concentration less than 18 mg%.

Based on the inclusion and exclusion criteria used, 110 newborns were qualified for further analysis in this research project. The neonates were classified into two groups: the meningitis group (*n* = 33) and the non-meningitis group (*n* = 77). The prenatal and postnatal period was also analyzed in both groups (Table 1). The population of children studied in this study included newborns born between weeks 23 and 42 of gestation with a birth weight from 750 to 2960 g. Pregnancy complications in mothers of newborns from both groups (gestational diabetes mellitus, urinary tract infection, performance of invasive procedures, colonization of the genital tract with Streptococcus agalactiae, premature rupture of the fetal bladder ≥ 18 h, chorioamnionitis, and lack of due prenatal) care were analyzed.

Determinations of CSF parameters were performed at the Laboratory of the University Clinical Hospital No. 2 PUM in Szczecin using a Cobas analyzer. Determination of procalcitonin concentration in ng/mL: using a Cobas analyzer sandwich assay, an in vitro test was performed to quantify procalcitonin in CSF. The analyzer automatically calculates the concentration in each sample in ng/mL. Samples were centrifuged before procalcitonin determination. Determination of lactate concentration in mmol/L: using a Cobas analyzer by colorimetric method, an in vitro test was performed for the quantification of lactate in CSF on Roche/Hitachi Cobas C. Samples were centrifuged before lactate determination.

We compared the concentration values of routinely determined CSF parameters and additional biochemical parameters, such as procalcitonin and lactate concentrations, determined in CSF in the meningitis and non-meningitis groups.

The relative risk (OR) of meningitis was also determined for the biochemical parameters studied and the routinely determined cytosis and protein concentration in CSF using the logistic regression method.

Using statistical analysis methods, ROC curves were drawn and cutoff points for lactate and procalcitonin were determined. This allowed us to determine the diagnostic usefulness of these parameters in the diagnosis of CSF.

Consent to perform the study in the Department of Neonatal Diseases was obtained from the Bioethics Committee of the Pomeranian Medical University in Szczecin (KB-0012/23/02/2021 of 23 February 2021). The study followed the Declaration of Helsinki (2013).

Written informed consent was obtained from parents of newborns.

### Statistical Analysis

The statistical analysis was conducted using Statistica 13 software.

Our study with 33 meningitis neonates and 77 non-meningitis controls had sufficient statistical power to detect with 80% probability true differences between groups corresponding to 0.6 SD (standard deviation) of the analyzed parameters, and OR (odds ratio) 0.29 or 3.52 for association of the parameters with meningitis in logistic regression model.

Nominal variables were presented as counts (percentage) and compared between groups using Fisher’s exact 2-tailed test. The Mann–Whitney U test was used to compare quantitative and rank variables between groups since in most cases the distributions of the parameters of measurable clinical characteristics were significantly different from normal distribution (*p* < 0.05, the Shapiro–Wilk test). Quantitative variables are presented as the mean ± standard deviation (SD) and/or median [interquartile range (IQR), Q1–Q3]. The sensitivity and specificity of the selected parameters for the diagnosis of meningitis were analyzed using the Receiver Operating Characteristics method (ROC), determining the proposed cutoff point based on the maximization of the Youden index. As an overall measure of the diagnostic performance of individual parameters, the value of the area under the ROC curve (AUC) was used. To evaluate the association of the selected parameters with meningitis, the bivariate logistic regression method was used,, and a subjecting the parameters, if necessary, to a logarithmic transformation normalizing their distribution. A *p* value of less than 0.05 was considered to be statistically significant.

## 3. Results

The median (IQR) age of lumbar puncture was 5 (2–14) days of life.

Both groups were comparable in terms of baseline characteristics (Table 1).

In the course of the present study, it was shown that symptoms such as seizures (*p* < 0.05) and muscle tone abnormalities in the form of hypertonia (*p* < 0.005) were significantly more frequent in the meningitis group compared to their frequency in the non-meningitis group. Importantly, the groups did not differ in terms of gestational duration and neonatal weight, but they differed significantly in APGAR scores obtained at 1 and 5 min of life (Table 1). Bacteriological cultures CSF were positive in 8 (24.2%) patients. We compared the concentrations (Median, IQR) of procalcitonin, lactate, glucose, protein, and pleocytosis in CSF in the meningitis and the non-meningitis group (Table 2).

**Figure 1:**
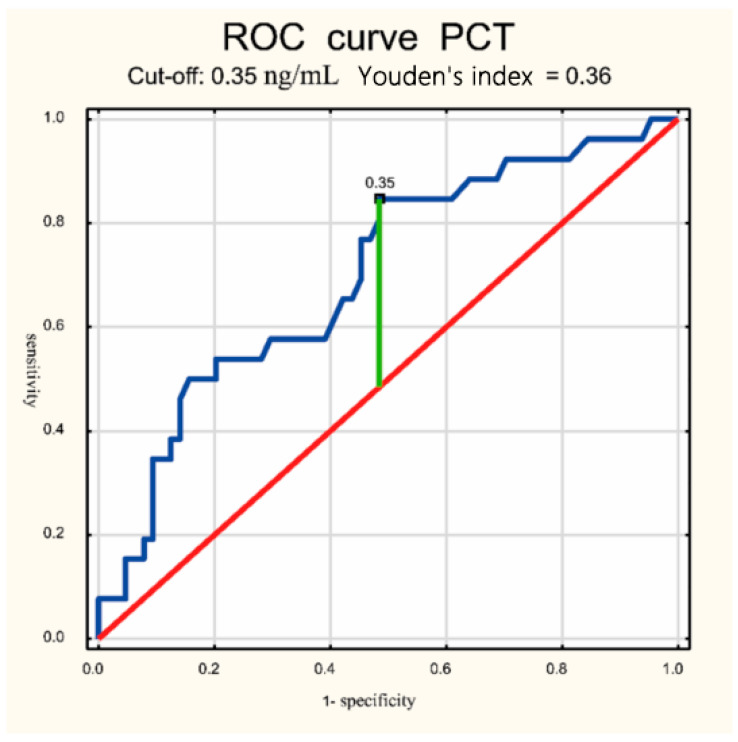
Receiver operating curve for CSF PCT for diagnosis of meningitis. CSF, cerebrospinal fluid; PCT, procalcitonin.

**Figure 2:**
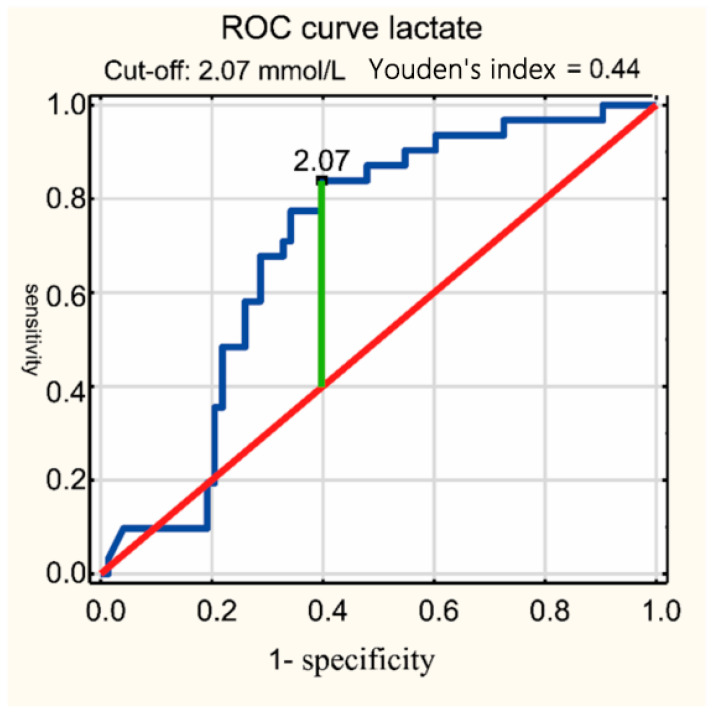
Receiver operating curve for CSF lactate for diagnosis of meningitis. CSF, cerebrospinal fluid.

The median CSF PCT level was significantly higher in the meningitis group compared to non-meningitis 0.93 (0.39–1.59) vs. 0.34 (0.195–0.74) ng/mL, respectively, *p* < 0.000001. The median CSF lactate level was significantly higher in the meningitis group compared to non-meningitis 3.1 (2.27–3.96) vs. 1.78 (1.38–3.19) mmol/L, respectively, *p* < 0.001. For the CSF biochemical parameters analyzed, cutoff points were established for the concentration of these parameters to make a diagnosis of meningitis: for lactate ≥ 2.07 mmol/L, the sensitivity was 84%, specificity 60%; for PCT ≥ 0.35 ng/mL, the sensitivity was 82%, and specificity 52% (Table 3). In Figure 1, the ROC curve was drawn. Analysis using ROC curves showed that lactate concentration in CSF differentiated the group of newborns with meningitis from the non-meningitis group at AUC = 0.7; *p* < 0.001. In Figure 2, the ROC curve was drawn. Analysis using ROC curves showed that lactate concentration in CSF differentiated the group of newborns with meningitis from the non-meningitis group at AUC = 0.701; *p* < 0.0001.

It was found that adding CSF PCT or CSF lactate concentrations to the routinely determined protein concentration (Table 4 and Table 5) or to pleocytosis value (Table 6 and Table 7) in CSF did not significantly increase the odds ratio for the diagnosis of meningitis in newborns. None of such two-factor models had *p* < 0.05 for the other biochemical parameters.

## 4. Discussion

The significance of CSF PCT in neonatal meningitis has not been widely studied to date. The topic has been addressed in only a few publications involving a small number of cases. With the exception of the study performed by Reshi et al. [23] on 75 neonates with meningitis, they were conducted on a small group of neonates diagnosed with meningitis, as 18 in Dutta et al. [24], 29 in Nagaraj et al. [25], and 17 in Rajial et al. [26]. The present study analyzed a group of 33 neonates with and 77 without meningitis. In the course of this study, it was shown that CSF PCT (median, IQR) was significantly higher in the meningitis group compared to the non-meningitis group: 0.93 (0.39–1.59) vs. 0.34 (0.195–0.74) ng/mL (*p* < 0.003). These results are consistent with those of previous studies in neonates by Reshi et al. [23], Dutta et al. [24], Rajial et al. [26], and Nagaraj et al. [25]. In this study, the CSF PCT in both groups was higher than in previously published works, as Reshi et al. found a median (Q1, Q3) of 0.47 (0.38–0.88) vs. 0.26 (0.21–0.28) ng/mL (*p*< 0.001) [23]; in Nagaraj et al. 0.194 (0.034–0.534) vs. 0.012 (0.012–0.012) ng/mL (*p* < 0.001) [25]; and in Rajial et al. 0.31 vs. 0.11 ng/mL (*p* < 0.001) [26]. Only in the study of Dutta et al. were the mean values of CSF PCT in the meningitis group slightly higher (0.967 ng/mL) and slightly lower in the non-meningitis group (0.318 ng/mL (*p* < 0.028)) than those presented in this work [24]. The reasons for the higher PCT values found in the study may be due to differences in various kinds regarding the study group compared to groups evaluated by other researchers. Also, the timing of the test from the time of neonatal deterioration may have influenced the higher CSF PCT levels in this study. The median age at the time of lumbar puncture was 5 days, and previous antibiotic exposure was not an exclusion criterion for the study; 80% of the newborns included in this study were on antibiotic therapy. Rajial et al. used birth age as an inclusion criterion and included neonates born above 34 completed weeks of gestation, and excluded neonates who were on antibiotic therapy [26]. Reshi et al. recruited only neonates up to 28 days of age and with a higher weight (median 2800 g), excluded neonates who had received antibiotics before lumbar puncture, and made adjustments for traumatic pleocytosis [23]. Dutta et al. qualified neonates between 0 and 56 days of life, and excluded neonates who had received at least one dose of antibiotics before lumbar puncture and with a traumatized CSF sample [24]. Nagaraj et al., on the other hand, did not include neonates who had received antibiotics for more than 2 days before the lumbar puncture [25]. In this study, the cutoff point for CSF PCT as a marker of neonatal meningitis ≥ 0.35 ng/mL had a sensitivity of 82%, specificity of 52%, 95% confidence interval 0.58–0.82, and the positive and negative predictive value was 54% and 20%, respectively. The obtained sensitivity value for PCT CSF as a marker of meningitis is high and specificity is lower, which limits the usefulness of this indicator as a single marker of meningitis. Reshi et al. proposed a cutoff point for CSF PCT very similar, as it was ≥0.33 ng/mL (sensitivity 92%, specificity 87%, 95% confidence interval 0.887–0.964, positive and negative predictive value 85.2% and 93%) [23]. Other cutoff points for CSF PCT have been described by subsequent investigators. Rajian et al. showed that the cutoff point value ≥ 0.2 ng/mL had a sensitivity of 95.2% and specificity of 96%, a 95% confidence interval of 0.98–1.00, and a positive and negative predictive value of 95.45% and 100%, respectively [26]. Nagaraj et al. found that with a cutoff value of 0.120 ng/mL of CSF PCT sensitivity of 83%, a specificity of 84% was reported [25]. In another study, Dutta et al. found that the optimal cutoff for CSF PCT was 0.554 ng/mL; at this value of PCT concentration in CSF, the sensitivity was 92%, specificity 87%, with a 95% confidence interval 0.487–0.826 [24]. Based on the results, there was a significant association between meningitis and PCT concentration values in CSF. However, the diagnostic efficacy of CSF PCT is moderate and is not superior to the standard tests used to date, i.e., CSF pleocytosis and CSF protein concentration. This conclusion is consistent with the results of Dutta et al. [24]. On the other hand, Reshi et al. [23], Rajian et al. [26], and Nagraj et al. [25] found that procalcitonin in CSF has diagnostic efficacy similar to the standard parameters assessed in cerebrospinal fluid.

The present study showed that lactate concentration (median, Q1, Q3) in CSF was significantly higher in the meningitis group compared to the non-meningitis group, and was respectively: 3.1 (2.27–3.96) vs. 1.78 (1.38–3.19) mmol/L (*p* < 0.001). This result is consistent with the results of previous studies conducted in different age groups. Zhao et al. showed that the lactate concentration (median) in the group meningitis was higher compared to that of the non-meningitis group, and was respectively: 4.2 mmol/L vs. 1.3 mmol/L (*p* < 0.05) [27]. Yadav et al. reported significantly higher mean lactate levels among adult patients with meningitis compared to the group without this diagnosis (6.6 ± 4.8 mmol/L vs. 2.6 ± 1.2 mmol/L, *p* < 0.001) [18]. Similarly, Singh et al. found that lactate levels were 6.25 ± 5.05 vs. 1.29 ± 0.60 (*p* < 0.001), respectively [19].

In this study at a cutoff of 2.07 mmol/L, CSF lactate had a sensitivity of 84% and specificity of 60% in the diagnosis of meningitis (AUC = 0.701). The obtained sensitivity value for lactate as a marker of meningitis is high and specificity is lower, which limits the usefulness of this indicator as a single marker of meningitis. Zhao et al. reported an optimal cutoff point of 2.2 mmol/L, with a PPV of 72.7% and an NPV of 100.0% for the diagnosis of neonatal meningitis [27]. Yadav et al. in a study conducted in adults presented a cutoff point of >2 mmol/L with AUC = 0.81, (*p* ≤ 0.001) [18]. In a group of pediatric patients, Nazir et al. [14] showed a cutoff point of 2.4 mmol/L, and Mekitarian Filho et al. [21] presented a cutoff point of 3.0 mmol/L with AUC = 0.96. The cutoff values for CSF lactate concentration for the diagnosis of meningitis still do not have reference standards, with various authors using cutoff values in the range of 2.1–4.4 mmol/L, so the cutoff value reported in this study is within the generally accepted range [22]. A similarly high sensitivity for lactate CSF for the diagnosis of meningitis is found in earlier studies in adults and children. Yadav et al. [18] showed that CSF lactate concentration at a cutoff point of 2.7 mmol/L for the diagnosis of FMS had a sensitivity of 82.6%, while Mekitarian Filho et al. [21] showed that at a cutoff point of 3.0 mmol/L, the sensitivity was 95%. On the other hand, Singh et al. [19] and Nasir et al. [20] report that lactate concentration in CSF shows up to 100% sensitivity, but they did not provide a cutoff point value. A meta-analysis by Huy et al. showed that CSF lactate concentration ≥ 3.5 mmol/L was associated with a high sensitivity of 96–99% and specificity of 88–94% in diagnosing bacterial meningitis [22]. A meta-analysis by Xiao et al. also reported the same results: sensitivity of 92% and specificity of 88% [28]. According to the information available in the literature, the determination of lactate concentration in the CSF in the diagnosis of meningitis should take place before the administration of antibiotics, since exposure to antibiotics can reduce its sensitivity [29]. However, in this study, 80% of patients, and in the study by Yadav et al. [18], more than 90% of patients, received an antibiotic prior to lumbar puncture, and the sensitivity of determining the lactate concentration in the cerebrospinal fluid established for the diagnosis of meningitis remained good.

This study has several limitations. It was a monocentric study with a relatively small number of patients. However, despite the small number of patients, we still found a significant difference between groups, with confidence intervals that are sufficiently narrow to allow precise determination of between-group differences. We measured PCT and lactate in CSF at one point in time only. We did not know at what moment after infection the peak levels were reached or when the PCT and lactate levels started to decrease again. The cost of performing CSF with PCT and lactate level vs. routinely determined CSF parameters was twice as high (116 zl vs. 60 zl). As the study was designed to collect data on CSF PCT and lactate prospectively followed by an analysis, PCT and lactate levels were not available for all patients in the meningitis and non-meningitis groups. A further multi-center study, with larger study groups on this subject, is needed to assess the usefulness of these markers in clinical practice.

To conclude, PCT and lactate in CSF were found to be significantly elevated in cerebrospinal fluid in neonates with meningitis compared to the non-meningitis group. These results show a good sensitivity and negative predictive value, but they need to be seen only as a measure for easier interpretation of the outcomes. Individually, neither of the two additional CSF biochemical parameters analyzed provides a significant advantage over the routinely determined pleocytosis and protein concentration in CSF.

Establishing cutoff points for PCT and lactate in CSF is particularly important for making therapeutic decisions in situations where the interpretation of classical parameters in CSF is questionable.

## 5. Conclusions

Concentrations in CSF of PCT and lactate in neonates with meningitis are significantly higher than in the non-meningitis group. None of the biochemical indicators studied met the criteria for a marker for the diagnosis of meningitis as a single indicator. In combination with other validated clinical prediction models, CSF lactate and PCT can assist with clinical decision making for newborns with meningitis.

## Figures and Tables

**Table 1:** Comparison of baseline characteristics of meningitis and no meningitis group.

Variables	Group Meningitis (*n* = 33)	Group Non-Meningitis (*n* = 77)	*p*-Value
Male, *n* (%)	24 (72.73)	50 (64.94)	>0.5
Female, *n* (%)	9 (27.27)	27 (35.06)	
Gestational age (wk), mean ± SD	31.2 ± 5.7	32.7 ± 5.0	<0.16
Birth weight (g), median (IQR)	1560 (750–2960)	1880 (1130–2760)	<0.32
Apgar score at 1 min, median (IQR)	6 (4–8)	7 (6–9)	<0.05
Apgar score at 5 min, median (IQR)	7 (6–8)	8 (7–9)	<0.02
EOS *n* (%), LOS *n* (%)	29 (87.9), 16 (48.5)	63 (81.8), 26 (33.7)	<0.6/<0.2
Clinical features, *n* (%)			
Hypotonic	17 (51.5)	64 (83.1)	<0.001
Hypertonia	9 (27.3)	5 (6.5)	<0.005
Seizures	5 (15.2)	3 (3.9)	<0.05
Abnormal body temperature	16 (48.5)	50 (64.9)	<0.1
Poor feeding	13 (39.4)	20 (25.9)	<0.2
Irritability	16 (48.5)	42 (54.6)	<0.6
Lethargy	16 (48.5)	42 (54.6)	<0.7
Apnea	16 (48.5)	37 (48)	=1.0

Abbreviations: EOS, early-onset sepsis; IQR, interquartile range; LOS, late-onset sepsis; SD, standard deviation.

**Table 2:** Comparison of CSF parameters, PCT CSF, lactate CSF levels in neonates with meningitis and no meningitis.

CSF Parameters	Group Meningitis (*n* = 33)	Group Non-Meningitis (*n* = 77)	*p*-Value
protein (mg/dL), median (IQR), *n*	214 (143.9–313.6), 33	108.4 (88.8–133.3), 77	<0.00001
sugar (mg/dL), median (IQR), *n*	52 (35–65), 33	52.5 (46–64), 77	<0.1
pleocytosis (cells per mm^3^), median (IQR), *n*	176.5 (82.5–593.5), 33	9 (5–16), 77	<0.00001
PCT (ng/mL), median (IQR), *n*	0.93 (0.39–1.59), 26	0.34 (0.195–0.74), 64	<0.003
Lactate (mmol/L), median (IQR), *n*	3.1 (2.27–3.96), 31	1.78 (1.38–3.19), 73	<0.001

Abbreviations: CSF, cerebrospinal fluid; IQR, interquartile range; PCT, procalcitonin.

**Table 3:** Diagnostic accuracy of the parameters studied in CSF for the diagnosis of meningitis.

	Lactate (mmol/L)	Procalcitonin (ng/mL)
AUC	0.701	0.7
95% CI of AUC	0.599–0.804	0.58–0.82
*p* value of AUC	<0.0001	<0.001
Optimal cutoff	2.07	0.35
Sensitivity (%)	84	85
Specificity (%)	60	52
PPV	0.47	0.42
NPV	0.90	0.892
+LR	2.11	1.75
−LR	0.27	0.298

Abbreviations: AUC, area under the receiver operating characteristic curve; CSF, cerebrospinal fluid; −LR, negative likelihood ratio; +LR, positive likelihood ratio; NPV, negative predictive value; PPV, positive predictive value; 95% CI, 95% confidence intervals.

**Table 4:** Summary of the odds ratio of meningitis diagnosis in newborns using logarithm of CSF protein concentration and logarithm of CSF lactate concentration by two-factor logistic regression method.

Regression with Two Independent Variables	OR	95% CI	*p*-Value
Log CSF protein and Log CSF lactate	20.5; 1.64	5.6–74.9; 0.90–2.96	<0.00001; >0.09

Abbreviations: CSF, cerebrospinal fluid; Log, logarithm; OR, odds ratio; 95% CI, 95% confidence intervals.

**Table 5:** Summary of the odds ratio of meningitis diagnosis in newborns using logarithm of CSF protein concentration and logarithm of CSF PCT concentration by two-factor logistic regression method.

Regression with Two Independent Variables	OR	95% CI	*p*-Value
Log CSF protein and Log CSF PCT	12.1; 1.5	3.23–44.9; 0.83–2.69	<0.0002; >0.17

Abbreviations: CSF, cerebrospinal fluid; Log, logarithm; OR, odds ratio; PCT, procalcitonin; 95% CI, 95% confidence intervals.

**Table 6:** Summary of the odds ratio of meningitis diagnosis in newborns using logarithm of CSF pleocytosis value and logarithm of CSF Lactate concentration by two-factor logistic regression method.

Regression with Two Independent Variables	OR	95% CI	*p*-Value
Log CSF pleocytosis and Log CSF lactate	11.7; 0.88	4.01–34.04; 0.33–2.34	<0.00001; >0.8

Abbreviations: CSF, cerebrospinal fluid; Log, logarithm; OR, odds ratio; 95% CI, 95% confidence intervals.

**Table 7:** Summary of the odds ratio of meningitis diagnosis in newborns using logarithm of CSF pleocytosis value and logarithm of CSF PCT concentration by two-factor logistic regression method.

Regression with Two Independent Variables	OR	95% CI	*p*-Value
Log CSF pleocytosis and Log CSF PCT	9.35; 1.63	3.3–26.4; 0.66–402	<0.00001; >0.3

Abbreviations: CSF, cerebrospinal fluid; Log, logarithm; OR, odds ratio; 95% CI, 95% confidence intervals.

## Data Availability

Data are available upon request.
